# Supplementary figures and images for: Species Boundaries and Molecular Markers for the Classification of 16SrI Phytoplasmas Inferred by Genome Analysis
Source: Front Microbiol. 2020 Jul 10;11:1531. doi: 10.3389/fmicb.2020.01531 (PMC7366425; doi:10.3389/fmicb.2020.01531)

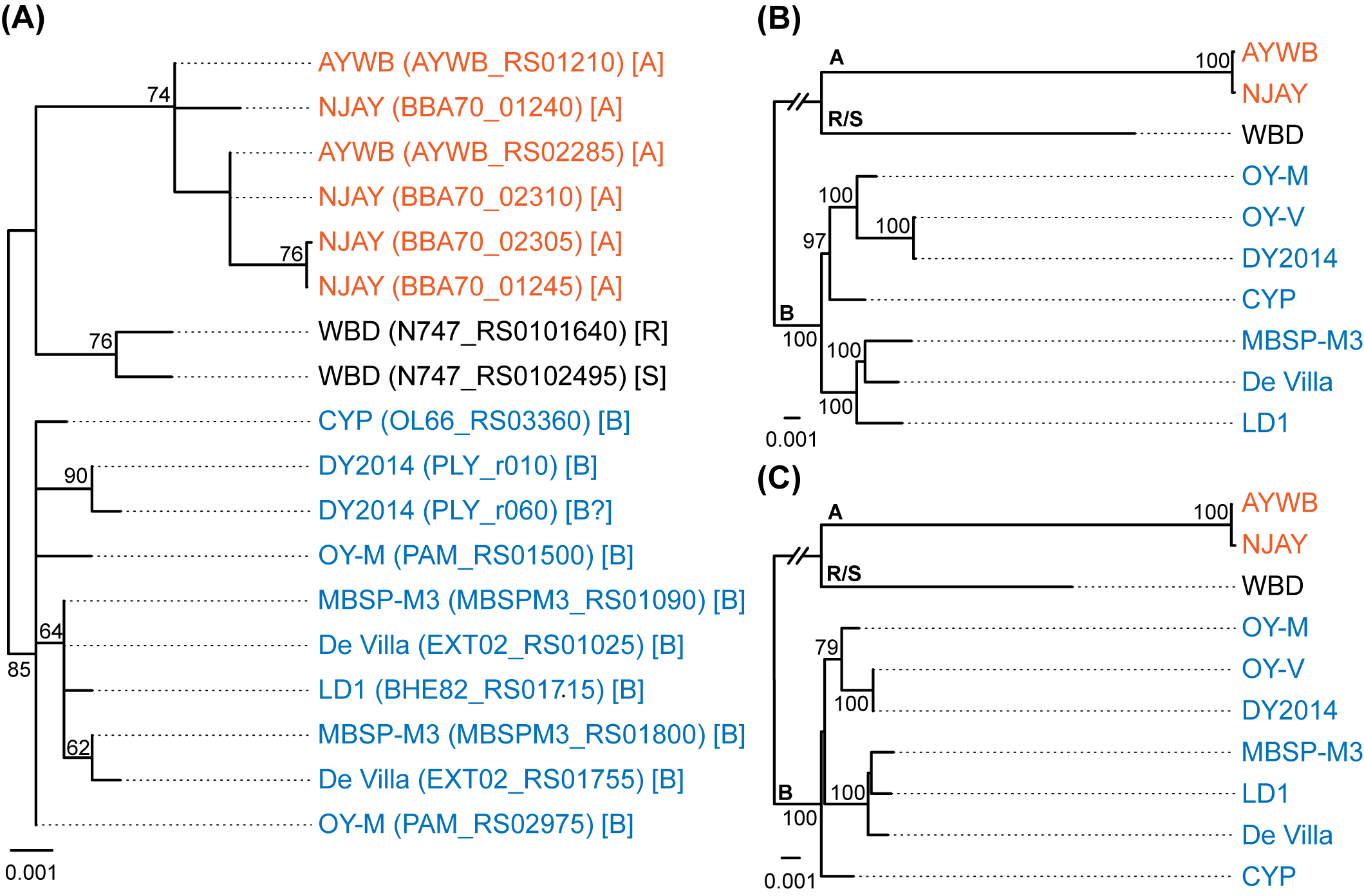

Supplement: FIGURE S1 — Maximum likelihood phylogenies. Based on Figure 1, the TW1 sequences were excluded prior to multiple sequence alignment. The numbers on the internal branches indicate the level of bootstrap support based on 1,000 resampling; only values ≥60% are shown. (A) 16S rRNA genes. The strain name, locus tag of the 16S rRNA gene (in parentheses), and the 16SrI subgroup classification (in square brackets) are labeled. A question mark ‘?’ in the subgroup classification indicates that the sequence is classified as a new subgroup, the existing subgroup with the highest similarity is provided. (B) The 303 single-copy coding genes shared by all strains. (C) 16S rRNA gene and the five markers developed in this study; see Table 2 for detailed information. [file Image_1.tif]

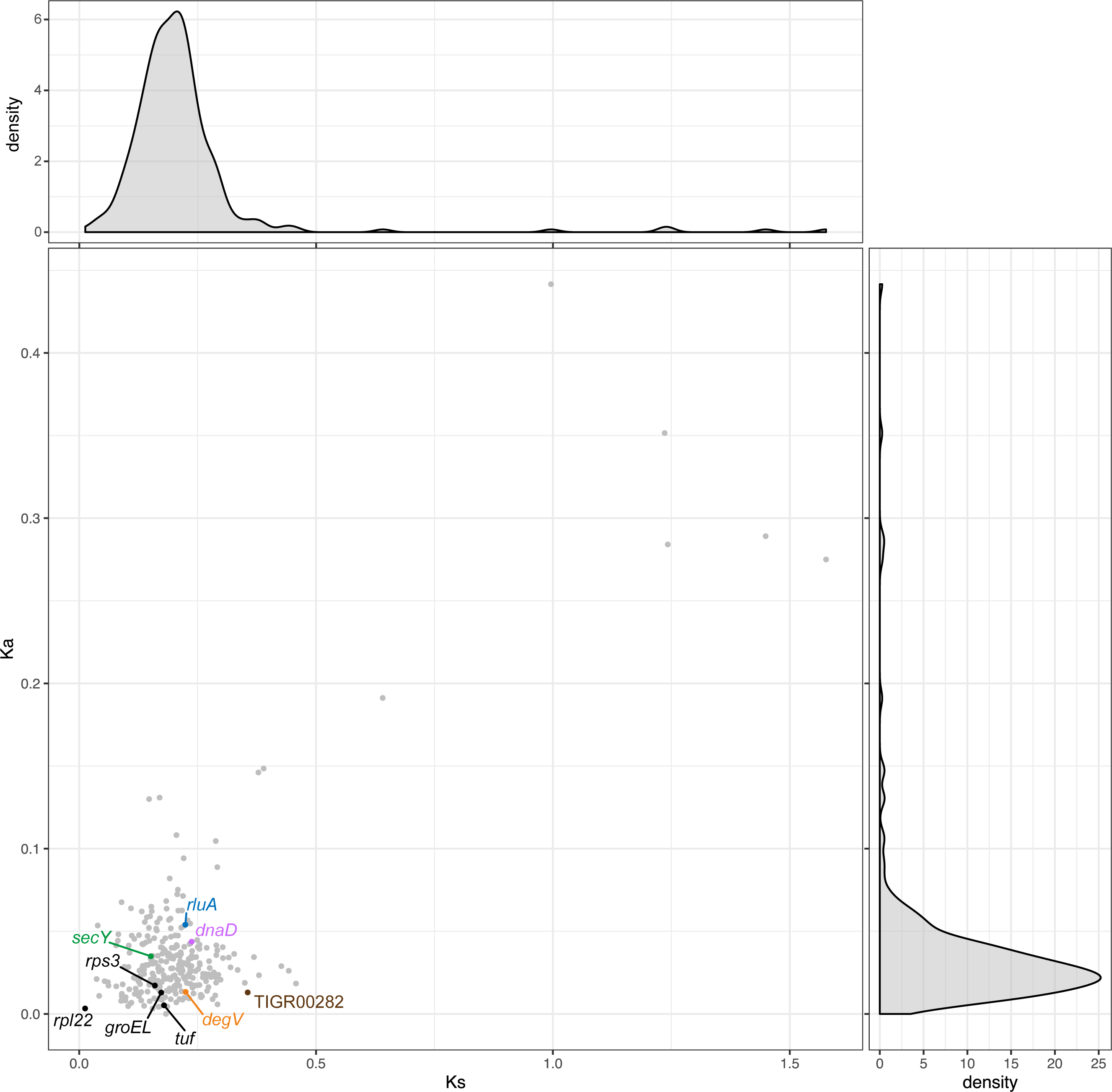

Supplement: FIGURE S3 — Distribution of substitution rates among those single-copy genes shared by the 11 phytoplasma genomes analyzed. The synonymous substitution rate (Ks) and non-synonymous substitution rate (Ka) were calculated based on the homologs found in AYWB and MBSP-M3. Among those 303 single-copy genes, nine were annotated as pseudogenes and were omitted from the substitution rate calculation. [file Image_3.tif]
