# Supplementary material for: Species Boundaries and Molecular Markers for the Classification of 16SrI Phytoplasmas Inferred by Genome Analysis
Source: Front Microbiol. 2020 Jul 10;11:1531. doi: 10.3389/fmicb.2020.01531 (PMC7366425; doi:10.3389/fmicb.2020.01531)
Supplement: FIGURE S2 — Multiple sequence alignments of selected marker genes. The primer sites are highlighted in orange. Shades of blue colors in the alignment indicate the levels of sequence conservation. [file Image_2.pdf]

(A) replication initiation protein DnaD

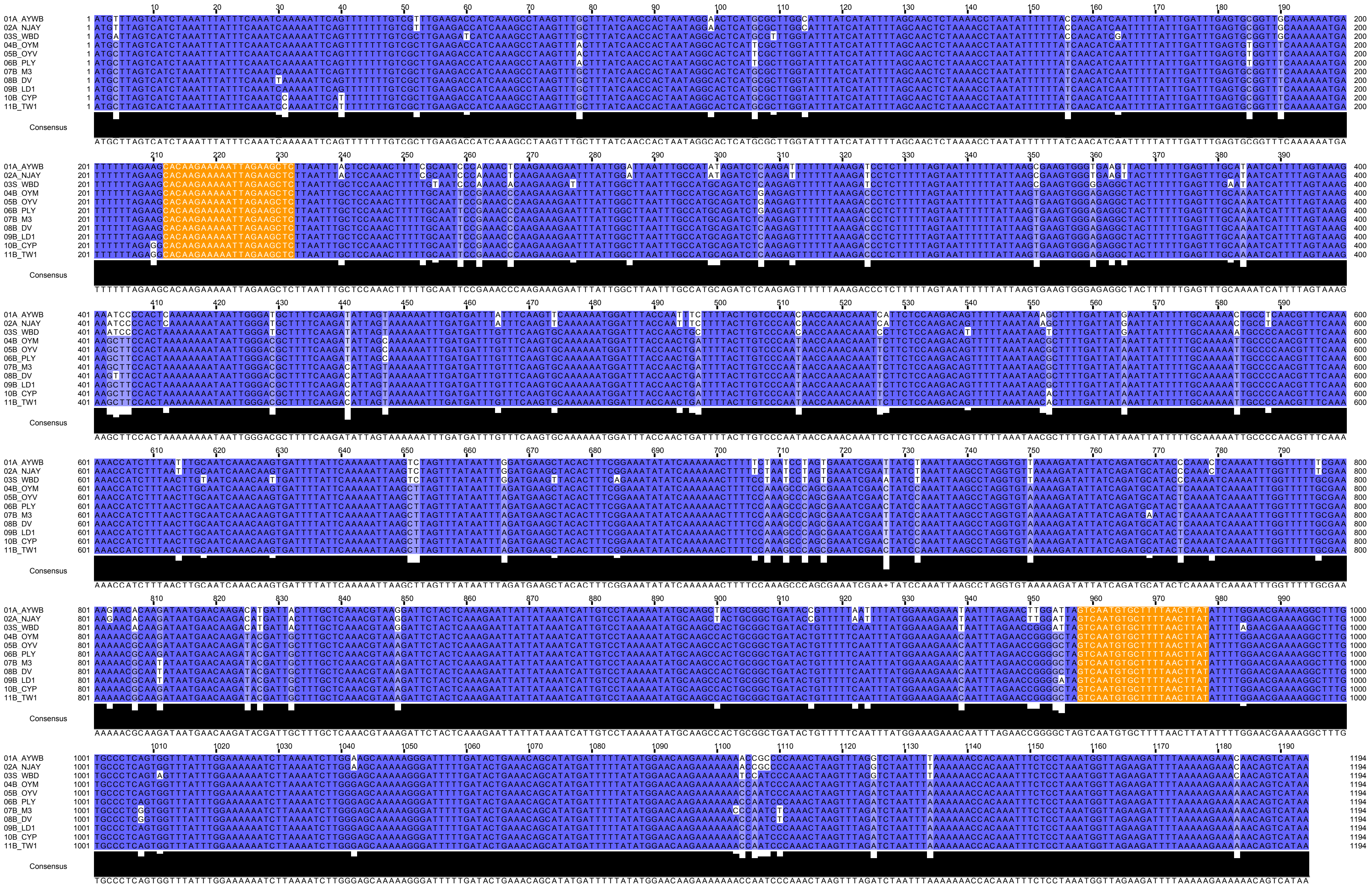

(B) DegV family protein

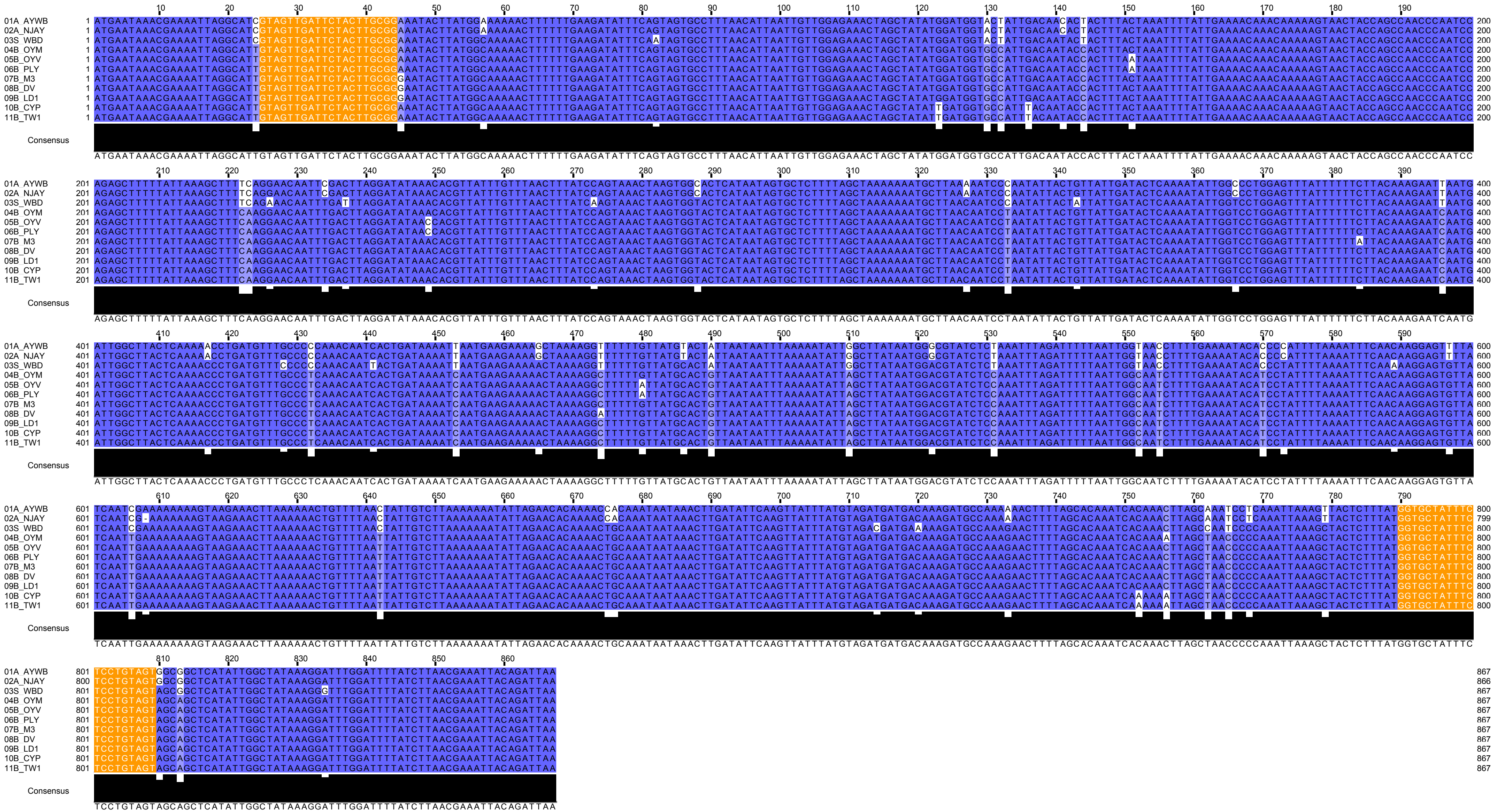

(C) TIGR00282 family metallophosphoesterase

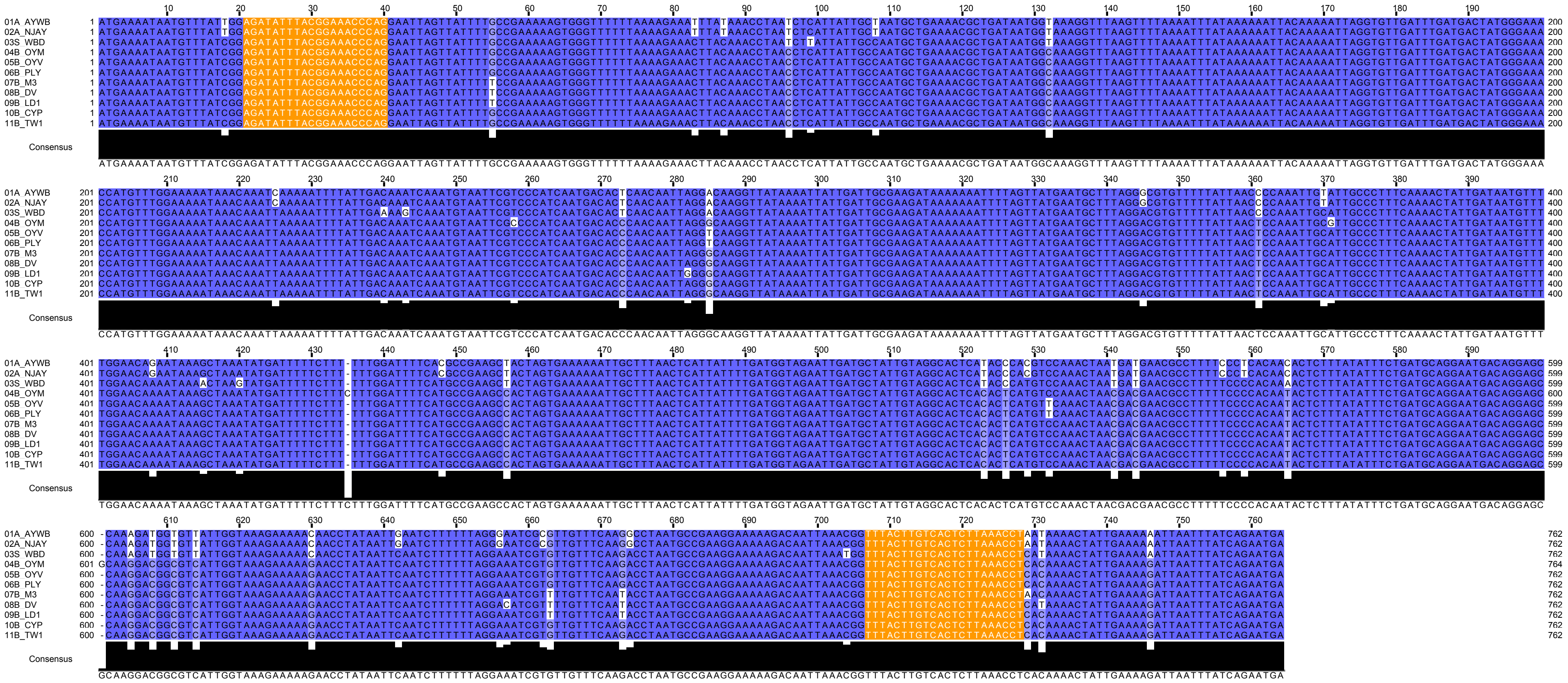

(D) preprotein translocase subunit SecY

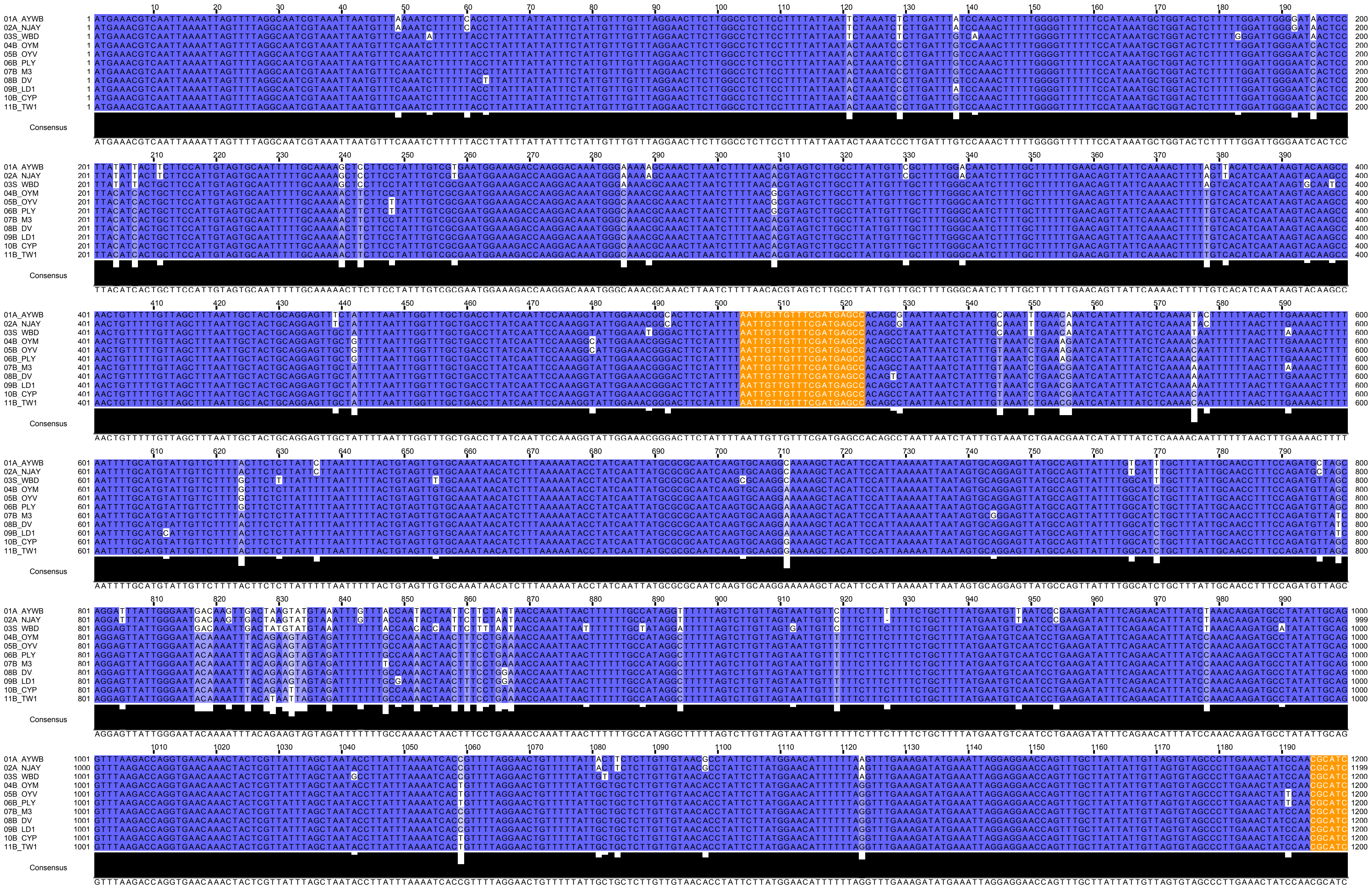

		1210 1220 1230 1240	
01A_AYWB	1201	AAAGCTACTGCCAACAAAAAAGAATATCAAAAATTATTTTAA	1242
02A_NJAY	1200	AAAGCTACTGCCAACAAAAAAGAATATCAAAAATTATTTTAA	1241
03S_WBD	1201	AAAGCTACTGCCAACAAAAAAGAATATCAAAAATTATTTTAA	1242
04B_OYM	1201	AAAGCTACTGCCAACAAAAAAGAATATCAAAAATTATTTTAA	1242
05B_OYV	1201	AAAGCTACTGCCAACAAAAAAGAATATCAAAAATTATTTTAA	1242
06B_PLY	1201	AAAGCTACTGCCAACAAAAAAGAATATCAAAAATTATTTTAA	1242
07B_M3	1201	AAAGCTACTGCCAACAAAAAAGAATATCAAAAATTATTTTAA	1242
08B_DV	1201	AAAGCTACTGCCAACAAAAAAGAATATCAAAAATTATTTTAA	1242
09B_LD1	1201	AAAGCTACTGCCAACAAAAAAGAATATCAAAAATTATTTTAA	1242
10B_CYP	1201	AAAGCTACTGCCAACAAAAAAGAATATCAAAAATTATTTTAA	1242
11B_TW1	1201	AAAGCTACTGCCAACAAAAAAGAATATCAAAAATTATTTTAA	1242
Consensus		AAAGCTACTGCCAACAAAAAAGAATATCAAAAATTATTTTAA	

(E) RluA family pseudouridine synthase

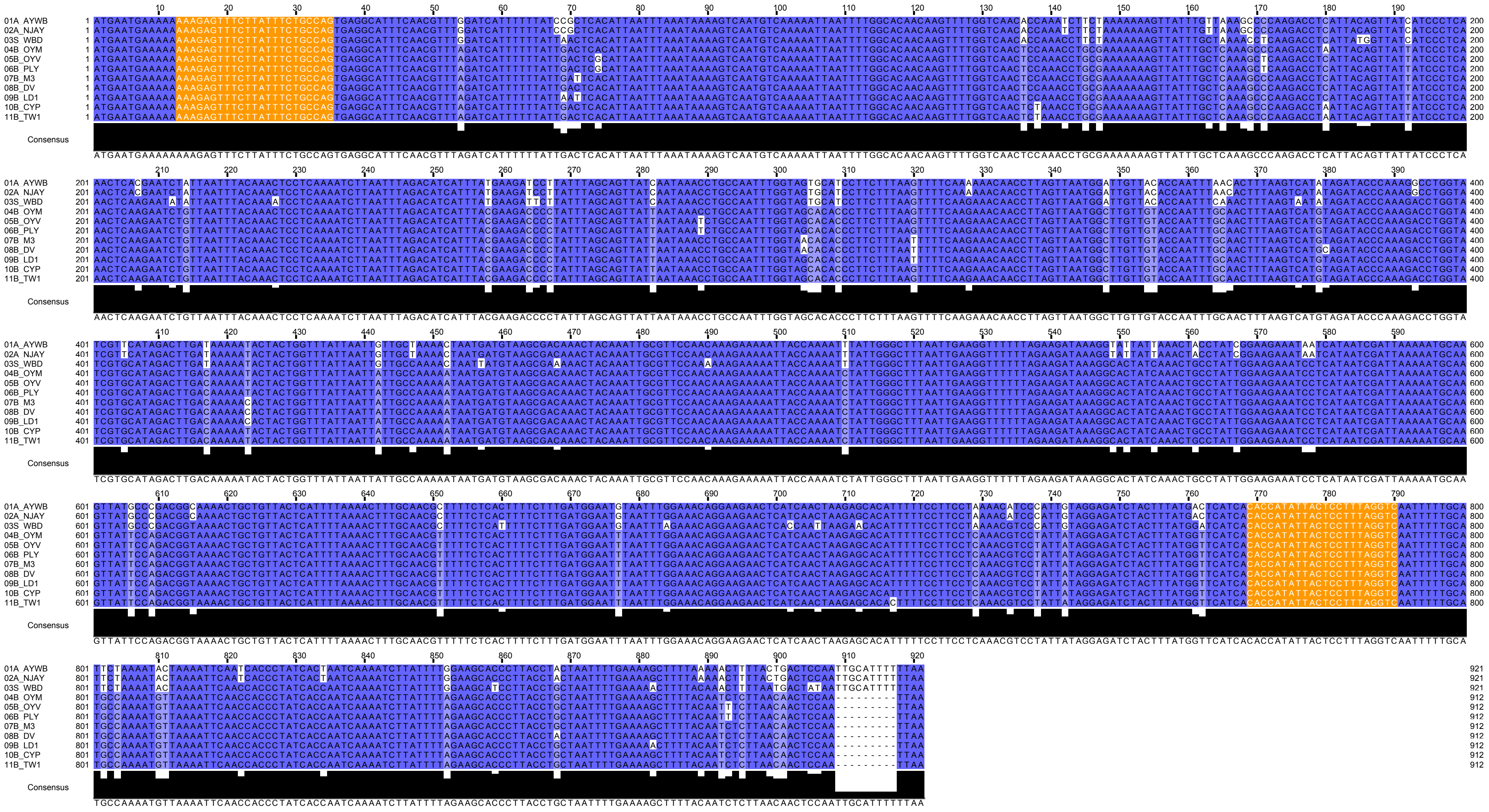

(R) 16S ribosomal RNA

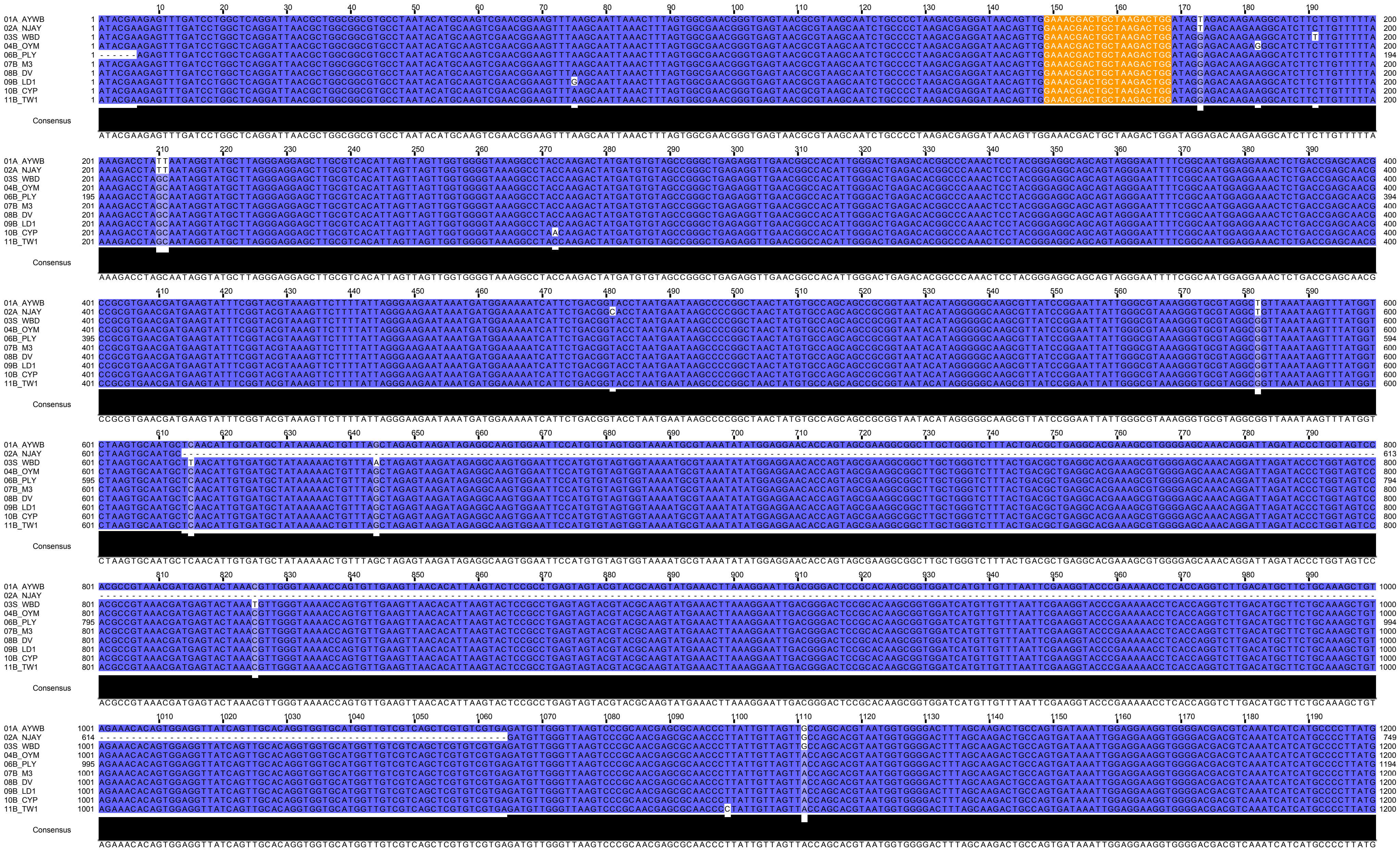

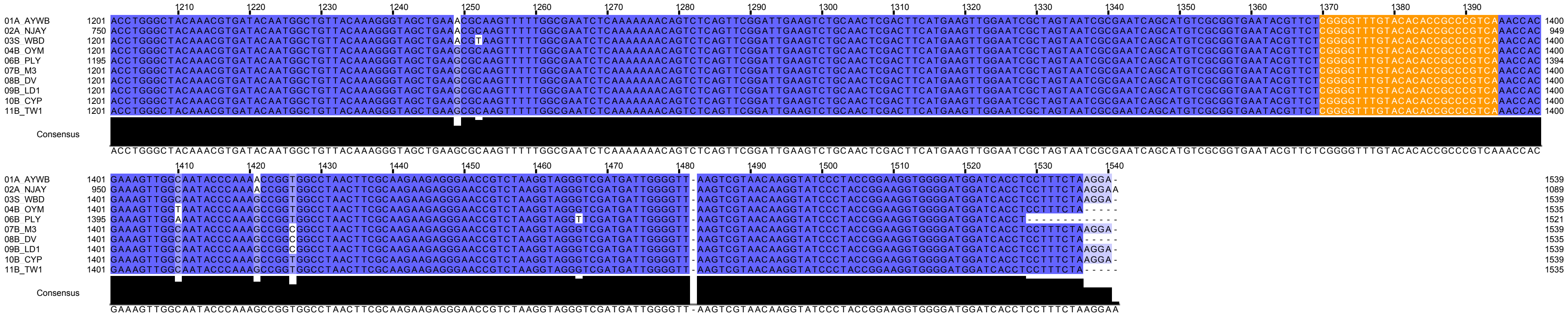
01A AYWB
02A NJAY
03S WBD
04B OYM
06B PLY
07B_M3
08B_DV
09B_LD1
10B CYP
11B_TW1
Consensus
ACCTGGGCTACAAACGTGATACAATGGCTGTTACAAAGGGTAGCTGAAGCGCAAGTTTTTGGCGAATCTCAAAAAAACAGTCTCAGTTCGGATTGAAGTCTGCAACTCGACTTCATGAAGTTGGAATCGCTAGTAATCGCGAATCAGCATGTCGCGGTGAATACGTTCTCGGGGTTTGTACACACCGCCCGTCAAACCAC
GAAAGTTGGCAATACCCAAAGCCGGTGGCCTAACTTCGCAAGAAGAGGGAACCGTCTAAGGTAGGGTCGATGATTGGGGTT-AAGTCGTAACAAGGTATCCCTACCGGAAGGTGGGGATGGATCACCTCCTTTCTAAGGAA
